# Remission of Kimura Disease With Carotid Hypervascularization After Cyclosporine Treatment

**DOI:** 10.5826/dpc.1002a30

**Published:** 2020-04-03

**Authors:** Severino Persechino, Armando Bartolazzi, Flavia Persechino, Antonella Tammaro, Sabatino Valente, Salvatore Raffa, Vincenzo Visco, Maria Rosaria Torrisi

**Affiliations:** 1 Dermatology Unit, Sant’Andrea Hospital, Sapienza University of Rome, Italy; 2 Histopathology Unit, Sant’Andrea Hospital, Sapienza University of Rome, Italy; 3 Department of Clinical and Molecular Medicine, Sant’Andrea Hospital, Sapienza University of Rome, Italy; 4 Ultrastructural Pathology Laboratory, Sant’Andrea Hospital, Sapienza University of Rome, Italy

**Keywords:** Kimura disease, cyclosporine, carotid hypervascularity, eosinophilia

## Introduction

Kimura disease was reported for the first time as “hyperplastic lymphogranuloma with eosinophilia” [1]. The cause of Kimura disease remains unknown, and reasons such as an allergic or unusual reaction to an unknown antigen or clonal aspecific lymphocyte proliferation have been considered. Some have hypothesized on the role of interleukins (IL-4, IL-5) and mast cells, regulating immunoglobulin E (IgE) synthesis and eosinophil infiltration.

## Case Presentation

**Figure 1:**
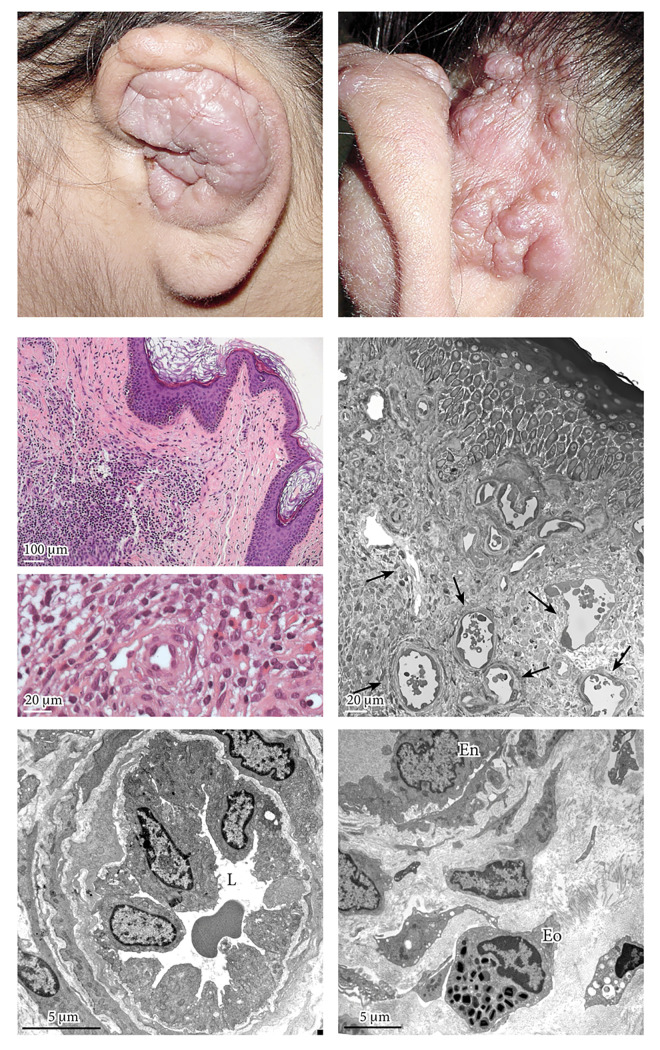
(A,B) Physical examination revealed an asymptomatic lymphadenomegaly of the left cervical region. The cutaneous lesions were nodules of 1–2 cm of maximum diameter, with a slightly hard consistency, with the upper layer slightly erythematous at the level of the auricle. (C–E) The histological findings from a biopsy of a lesion at the level of the left retroauricular region showed spongiosis of the epidermis and a remarkable proliferation of vascular structures of the derma (see black arrows in E) with hypertrophic endothelium protruding into the lumen and a strong perivascular inflammatory infiltrate with the presence of numerous eosinophil cells. (C,D) H&E staining. (E) Toluidine blue semithin section. (F,G) Ultrastructural analysis shows at the level of the papillary and reticular dermis many vascular structures lined by endothelial cells that protrude into the lumen. (F,G) Transmission electron microscopy, uranyl acetate/lead citrate. Morgagni 268D Electron Microscopy, FEI Company, Hillsboro, OR. En = endothelial cell; Eo = eosinophil; L = lumen.

A 34-year-old Caucasian woman presented with erythematous lightly hard nodules of 1–2 cm of maximum diameter, at the level of the auricle (Figure 1, A and B). Physical examination revealed an asymptomatic lymphadenomegaly of the left cervical region.

The only value of interest was the elevated IgE concentration (total IgE 3,775 IU/mL vs normal value 260 IU/mL). Ultrasonography of the thyroid and lymph nodes of the neck revealed the presence of multiple swollen, hypoechoic, and hyperplastic lymph nodes at the submandibular, parotid, and left ear region, where the largest was 3 cm with a hypervascular hilum. The histological findings from a biopsy of a lesion at the level of the left retroauricular region showed spongiosis of the epidermis and a remarkable proliferation of vascular structures of the derma with hypertrophic endothelium protruding into the lumen and a remarkable perivascular inflammatory infiltrate with the presence of numerous eosinophil cells (Figure 1, C–E). Ultrastructural analysis shows at the level of the papillary and reticular dermis many vascular structures lined by endothelial cells in massive columnar shape that protrude into the lumen (Figure 1, F and G).

We performed an arteriogram of the left carotid, which showed a pathological hypervascularity with at least 1 main branch and 2 secondary branches of the carotid tree that supplied the angiomatous lesion of the upper two-thirds of the ear and at least 3 veins that drained quickly the contrast medium to the external jugular vein.

Clinical and histological data and elevated concentration of IgE allow us to confirm a case of Kimura disease.

Considering that the location of the disease did not allow a surgical approach as it would put at serious risk the anatomical and functional integrity of the left ear, and the failure of local and systemic interferon and corticosteroid therapy, we chose immunosuppressant therapy with cyclosporine (3.5 mg/kg/day).

**Figure 2:**
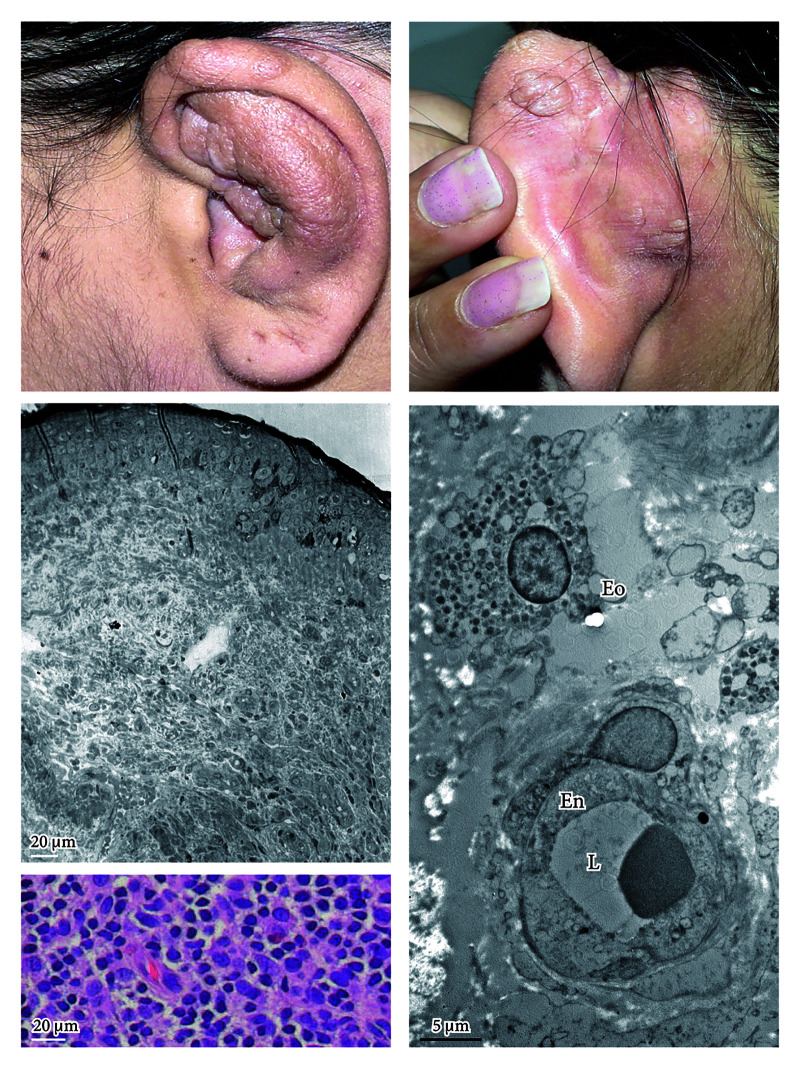
The patient was treated for 2 years without interruption, causing the regression of the cutaneous lesions at physical examination (see A and B) and reduction of the vascularization with eosinophilia at histological examination (see C and D). (C) Toluidine blue semithin section. (D) H&E staining. (E) Ultra-structural examination shows the persistence of eosinophilic infiltrate mostly localized around vascular structures. Transmission electron microscopy, uranyl acetate/lead citrate. Morgagni 268D Electron Microscopy, FEI Company, Hillsboro, OR. En = endothelial cell; Eo = eosinophil; L = lumen.

The patient was treated for 2 years, resulting in the regression of the cutaneous lesions (Figure 2, A and B) at physical examination and reduction of the vascularization with eosinophilia at histological examination (Figure 2, C and D). Ultrastructural examination shows the persistence of eosinophilic infiltrate mostly localized around vascular structures (Figure 2E). The most important differential diagnosis is angiolymphoid hyperplasia with eosinophilia (IALE). One of the differences between these two entities is the IgE concentration, which is high for Kimura disease and normal for IALE [2].

The total IgE amount was 875 UI/mL, with a reduction of vascularization at the arteriogram of the left carotid. Despite the risk of kidney disease associated with both Kimura disease and the use of cyclosporine, gingival hyperplasia was the only side effect in our patient. Nowadays the patient is followed every 6 months; 2 years after onset he had no recurrences.

## Conclusions

It seems relevant to highlight the unexpected effect of cyclosporine therapy, found by the arteriogram and never highlighted in current literature, namely the reduction of the neoangiogenesis and hypervascularization of the lesions as shown by the arteriography of the left carotid tree. This evidence could endorse the immunological mechanism for the pathogenesis of Kimura disease.
